# Alterations of Red Cell Membrane Properties in Nneuroacanthocytosis

**DOI:** 10.1371/journal.pone.0076715

**Published:** 2013-10-03

**Authors:** Claudia Siegl, Patricia Hamminger, Herbert Jank, Uwe Ahting, Benedikt Bader, Adrian Danek, Allison Gregory, Monika Hartig, Susan Hayflick, Andreas Hermann, Holger Prokisch, Esther M. Sammler, Zuhal Yapici, Rainer Prohaska, Ulrich Salzer

**Affiliations:** 1 Max F. Perutz Laboratories, Medical University of Vienna, Vienna, Austria; 2 Institute of Human Genetics, Technische Universität München, Munich, Germany; 3 Neurologische Klinik und Poliklinik, Ludwig-Maximilians-Universität, Munich, Germany; 4 Department of Molecular & Medical Genetics, Oregon Health & Science University, Portland, Oregon, United States of America; 5 Division of Neurodegenerative Diseases, Department of Neurology, Dresden University of Technology and German Centre for Neurodegenerative Diseases (DZNE), Dresden, Germany; 6 Institute of Human Genetics, Helmholtz Zentrum München, Neuherberg, Germany; 7 Neurology Department, Ninewells Hospital and Medical School, University of Dundee, Dundee, United Kingdom; 8 Division of Child Neurology, Department of Neurology, Istanbul Faculty of Medicine, Istanbul University, Istanbul, Turkey; 9 Departments of Pediatrics and Neurology, Oregon Health & Science University, Portland, Oregon, United States of America; Tufts University School of Medicine, United States of America

## Abstract

Neuroacanthocytosis (NA) refers to a group of heterogenous, rare genetic disorders, namely chorea acanthocytosis (ChAc), McLeod syndrome (MLS), Huntington’s disease-like 2 (HDL2) and pantothenate kinase associated neurodegeneration (PKAN), that mainly affect the basal ganglia and are associated with similar neurological symptoms. PKAN is also assigned to a group of rare neurodegenerative diseases, known as NBIA (neurodegeneration with brain iron accumulation), associated with iron accumulation in the basal ganglia and progressive movement disorder. Acanthocytosis, the occurrence of misshaped erythrocytes with thorny protrusions, is frequently observed in ChAc and MLS patients but less prevalent in PKAN (about 10%) and HDL2 patients. The pathological factors that lead to the formation of the acanthocytic red blood cell shape are currently unknown. The aim of this study was to determine whether NA/NBIA acanthocytes differ in their functionality from normal erythrocytes. Several flow-cytometry-based assays were applied to test the physiological responses of the plasma membrane, namely drug-induced endocytosis, phosphatidylserine exposure and calcium uptake upon treatment with lysophosphatidic acid. ChAc red cell samples clearly showed a reduced response in drug-induced endovesiculation, lysophosphatidic acid-induced phosphatidylserine exposure, and calcium uptake. Impaired responses were also observed in acanthocyte-positive NBIA (PKAN) red cells but not in patient cells without shape abnormalities. These data suggest an “acanthocytic state” of the red cell where alterations in functional and interdependent membrane properties arise together with an acanthocytic cell shape. Further elucidation of the aberrant molecular mechanisms that cause this acanthocytic state may possibly help to evaluate the pathological pathways leading to neurodegeneration.

## Introduction

Neuroacanthocytosis syndromes are a group of rare, neurodegenerative diseases that mainly affect children and young adults. This group of diseases shows similar clinical features such as dystonia, involuntary movements such as chorea and neurodegeneration of the basal ganglia [1,2]. Moreover, the appearance of spiky red blood cells (acanthocytes) with varying frequency (12% to 45% of red cells) is characteristic for these diseases [1]. Neuroacanthocytosis syndromes currently comprise four subtypes caused by mutations in distinct genes. Chorea-acanthocytosis (ChAc) relates to mutations in *VPS13A* [3,4], McLeod Syndrome (MLS) has mutations in *XK* [5], Huntington’s Disease-like 2 (HDL2) in *JPH3* [6], and panthotenate kinase-associated neurodegeneration (PKAN) in *PANK2* [7]. PKAN is also categorized among the Neurodegeneration with Brain Iron Accumulation (NBIA) syndromes characterized by iron deposition in the basal ganglia typically detected by brain MRI. NBIA syndromes share some clinical findings with NA and Huntington’s Disease [1]. NBIA syndromes are caused by mutations in a number of different genes [1], such as *PANK2*, responsible for PKAN, or *C19orf12* underlying mitochondrial membrane protein-associated neurodegeneration (MPAN) [8,9] In general, acanthocytosis has not been described in NBIA apart from PKAN where roughly 10% of PKAN patients are considered affected.

The reasons for the appearance of acanthocytes and their role in the diseases mentioned are unknown. Despite misshapen morphology, the red cells in NA and NBIA syndromes are functional. Patients are not affected by hypoxia or anemia. There are no lipid abnormalities in their blood either, which distinguish these syndromes from acanthocytosis conditions caused by liver cirrhosis, anorexia, or lipoprotein disorders. Moreover, coagulation dysfunctions are not reported for these patients.

Erythrocytes are adapted to rapid shape changes to allow the passage through the capillary system with its narrow spatial constraints. The elasticity and mechanical stability of these cells is provided by a actin-spectrin-based cytoskeletal network, which covers the cytoplasmic side of the membrane as a thick layer with a complex and dynamic architecture [10]. The cytoskeleton interacts with the abundant integral membrane proteins, band 3, glycophorin C and Glut1 via the adaptor proteins ankyrin, protein 4.1 and adducin. They organize the multi-component membrane attachment sites, known as “junctional complex” and “ankyrin complex” [11]. Cell shape regulation, that is the quick restoration of the discocytic shape after cessation of cell deforming conditions, is achieved by a complex interplay between cytoskeleton, membrane proteins and membrane lipids which is in essence still unresolved. Conformational changes and regulatory modifications (phosphorylation and/or proteolysis) of the band3 protein and associated cytoskeleton have been implicated to directly influence cell morphology [12–15]. Furthermore, various agents are known to induce erythrocyte shape changes *in vitro* and are classified as stomatocytogenic and echinocytogenic with respect to the type of erythrocytic shape change induced. Stomatocytic cells have only one, mouth-like invagination, whereas echinocytes have several spikes protruding from a more or less rounded cell body. Compared to acanthocytes, echinocytes have a higher number of spikes that are shaped regularly and distributed evenly at the cell surface. Drug-induced membrane distortions in stomatocytes and echinocytes are associated with the formation of endovesicles and exovesicles, respectively. Taking into account the amphipathic nature of the respective drugs, the bilayer-couple hypothesis by Sheetz and Singer [16] provides a good explanation for the induced changes in erythrocyte shape. The asymmetric distribution of the membrane phospholipids leads to preferential partitioning of positively and negatively charged amphipaths in the inner and outer membrane layer, respectively. The differential increase in the surface area of the respective layer accounts for the observed shape change where expansion of the cytoplasmic leaflet is associated with stomatocytosis and the increase of the outer leaflet with echinocytosis. Interestingly, the antidepressant drug chlorpromazine has been shown to convert acanthocytic cells of patients with abetalipoproteinemia [17], MLS [18] and ChAc [19] to a normal discocytic shape. Considering the cationic and amphipathic structure of this drug, this finding can be interpreted in line with the bilayer-couple hypothesis. Chlorpromazine obviously compensates a relatively increased ratio of the surface areas between outer and inner leaflet in acanthocytic cells and thereby restores the normal shape.

In this study, we aimed at the functional characterization of erythrocytes from ChAc, MLS and NBIA/PKAN patients with regard to specific membrane properties. Developing and applying flow cytometry-based assays for drug-induced endocytosis and lysophosphatidic acid-induced phosphatidylserine (PS) exposure and calcium uptake, we find abnormal responses in samples of patients that show a high proportion of acanthocytes. Interestingly, the behavior of PKAN blood samples with acanthocytes was similar to ChAc erythrocytes, whereas acanthocyte-free PKAN samples were rather indistinguishable from control cells. Thereby we propose and discuss an “acanthocytic state” of the red cell where altered functional membrane properties arise together with an acanthocytic cell shape. The manifestation of the acanthocytic state varies among the NA syndromes.

## Materials and Methods

### Blood samples

Blood samples were collected after obtaining written informed consent from patients and healthy donors. As part of the EMINA project, this study was approved by the ethics committee of Ludwig-Maximilians-Universität, Munich. Blood collection was performed in collaboration with the NA-Advocacy (London, UK) as well as with the Hayflick research repository (Oregon Health & Science University, in the USA). Freshly drawn blood in EDTA tubes from patients and healthy control donors (related or unrelated) was cooled and sent to Vienna over night. Part of the samples was fixed with 1.5% paraformaldehyde and 0.1% glutaraldehyde for morphological analysis. Then the samples were freshly used for the experiments or were frozen for later use. To that end, blood samples were centrifuged in falcon tubes for 15 minutes at 1900 rcf to separate red blood cells, buffy coat and blood plasm. Packed red blood cells were carefully mixed with an equal volume of 30% polyvinylpyrrolidone (PVP, average molecular weight 10,000, Sigma) solution in water. An empty styrofoam cuvette holder was filled with liquid nitrogen and the red blood cell-PVP mix was frozen drop wise using a pipette. Frozen drops were collected in pre-cooled cryotubes and stored in liquid nitrogen. To defrost red cells, the required number of frozen drops was added to pre-warmed (37°C) phosphate-buffered saline (PBS) and swirled. Cells were washed at least three times with PBS before use.

In total, 12 ChAc samples with 10 controls, 1 MLS sample with 1 control, 6 PKAN samples with acanthocytes (PKAN+) with 6 controls, 5 PKAN and 1 MPAN sample without acanthocytes (PKAN-/MPAN-) with 6 controls were tested (Table 1).

**Table 1:** Blood samples of patients, acanthocyte count and clinical information.

sample	sex	age	origin	acanthocytes	acanthocytes in control	molecular findings	mutations found	prominent clinical features
ChAc1	♀	33	DE	39,8%	20,2%	chorein missing	VPS13A, c.4282GC; c.7806GA,	chorea, epilepsy, tongue dystonia, dysarthria
ChAc2	♀	40	GB	21,1%	1,3%	chorein missing	VPS13A, 1208delAGAC; 7867C>T	chorea, epilepsy, tongue dystonia, dysphagia, dysarthria
ChAc3	♂	46	DE	24,8%	6,6%	chorein missing	VPS13A, c.8529_8530het_dupA; c.9078-2A>G,	epilepsy, dysarthria, symmetric parkinson syndrome, cognitive impairment
ChAc4	♂	48	GB	25,6%	11,1%	chorein missing	VPS13A, 237delT; 9429delAGAG	chorea, epilepsy, tongue biting, dysarthria
ChAc5	♀	39	GB	45,9%	3,1%	chorein missing	n/a	chorea, dysarthria
ChAc6	♂	26	DE	26,7%	7,0%	chorein missing	VPS13A, c.6059 delC, second mutation n/a	chorea, epilepsy
ChAc7	♂	21	DE	25,8%	11,3%	chorein missing	VPS13A, c.6059 delC, second mutation n/a	epilepsy
ChAc8	♀	43	DE	19,4%	10,4%	chorein missing	n/a	chorea, epilepsy, dysarthria, cognitive impairment
ChAc9	♂	53	GB	12,5%	3,6%	chorein missing	n/a	chorea
ChAc10	♀	47	ES	1,5%	3,5%	n/a	VPS13A, exon 54 deletion, homozygous	n/a
ChAc11	♂	45	ES	40,0%	3,5%	n/a	VPS13A, exon 54 deletion, homozygous	n/a
ChAc12	♂	38	ES	49,0%	3,5%	n/a	VPS13A, exon 54 deletion, homozygous	n/a
MLS1	♂	63	GB	33,3%	2,1%	Kx missing	XK, c. 1023G>A	profound orofacial dyskinesia, dysphagia, dysarthria, severe sensorimotor neuropathy with generalized muscle wasting, bedbound, pituitary macroadenoma on MR imaging, CK aemia.
PKAN+1	♂	7	DE	29,2%	0,7%	n/a	PANK2, c.1561GA p.G521R, second mutation n/a	n/a
PKAN+2	♂	12	TR	22,1%	1,5%	n/a	PANK2, c.628+2TG, homozygous	* generalized dystonia, bilateral mild rigidity, mild pyramidal signs, no walking since age 8
PKAN+3	♀	8	TR	42,3%	1,8%	n/a	PANK2, c.664CT, homozygous	* generalized dystonic and pyramidal signs prominent in lower limbs
PKAN+4	♂	15	TR	33,1%	2,3%	n/a	PANK2, c.664CT, homozygous	* generalized dystonic and pyramidal signs in the lower extremities
PKAN+5	♂	7	USA	17,5%	4,6%	n/a	PANK2, c.215insA, homozygous	n/a
PKAN+6	♂	9	TR	41,1%	12,8%	n/a	PANK2, c.1325_1328ATAG homozygous	* oromandibular and axial dystonia from age 7
MPAN-1	♀	14	TR	3,4%	6,0%	n/a	c19orf12, c.194GA p. G65E, homozygous	* dystonic movements in extremities, prominent in the limbs, pyramidal and cerebellar signs
PKAN-2	♂	33	TR	3,7%	6,5%	n/a	PANK2, c.1466TC; 1583C>T	very severe generalized dystonia, some pyramidal signs
PKAN-3	♀	27	USA	1,0%	2,9%	n/a	PANK2, c.1231GA; c.1255AG	n/a
PKAN-4	♂	25	USA	0,0%	3,2%	n/a	PANK2, c.1231GA; c.1255AG	n/a
PKAN-5	♀	25	USA	2,4%	1,9%	n/a	PANK2, c.987del; c.1253CT	n/a
PKAN-6	♀	17	USA	1,4%	2,6%	n/a	PANK2 (details n/a)	n/a

**Patient characteristics and acanthocyte count in patient samples and their respective controls**. Results of molecular and/or genetic analysis as well as clinical findings in the patients are given where available.

ChAc: Chorea Acanthocytosis, MLS: McLeod Syndrome. PKAN+: panthotenate kinase-associated neurodegeneration with acanthocytosis. PKAN- panthotenate kinase-associated neurodegeneration without acanthocytes. MPAN-1: mitochondrial membrane protein-associated neurodegeneration diagnosed patient, counted to the PKAN- group because of the lack of acanthocytes. Age of the patient at the time of blood sampling and code for country of sample origin are given. Findings from molecular and/or genetic diagnosis and clinical presentation of the patients are given. * denotes offspring of consanguinity marriage. n/a means detailed information is not available.

Please, note that patients ChAc6 and Chac7 are siblings, as are patients ChAc10-12 as well as patients PKAN+3 and PKAN+4 and patients PKAN-3 and PKAN-4.

Patients ChAc1 and ChAc2 correspond to patients 1 and 2, respectively, of Bader et al. [44]. ChAc4 is patient 4 of Dobson-Stone et al. [45]. ChAc6 and ChAc7 are patients 2 and 1, respectively, of Scheid et al. [46]. ChAc10-12 will be described in Velayos-Baeza et al. (manuscript in preparation).

### Microscopy

Fixed cells were suspended in PBS, dropped on microscopy slides, and allowed to settle down. Upon removal of the supernatant, cells were allowed to dry, fixed in pure methanol and stained with Diff Quick® (Labor+Technik, Salzburg, Austria) solutions following the manufacturer’s instructions. Cells were analyzed with Zeiss LSM Meta using the 63x oil objective and ZEN software. Pictures were taken and 200-300 cells were counted and classified as discocyte, misshapen cells or acanthocytes. Acanthocytic samples were identified by comparison with their respective control samples that were shipped under the same conditions.

### Drug-induced endovesiculation

Frozen erythrocytes (with 30% PVP in liquid nitrogen) were defrosted in pre-warmed PBS and washed with PBS containing 7.5 mM glucose three times. Cells were washed once in Hank’s Buffered Salt Solution (HBSS, 0.137 M NaCl, 5.4 mM KCl, 0.25 mM Na_2_HPO_4_, 0.44 mM KH_2_PO_4_, 1.3 mM CaCl_2_, 1.0 mM MgSO_4_, 4.2 mM NaHCO_3_) with 7.5 mM glucose and the volume was adjusted to a cell concentration of 2.5x10^9^ cells/ml. Fluorescein isothiocyanate (FITC) – dextran (FD70S, Sigma Aldrich) (100 mg/ml) was added to the cells to a final concentration of 10 mg/ml. For each sample, 4.5x10^7^ cells were taken from that preparation and stimulated with either chlorpromazine hydrochloride (Sigma Aldrich) at final concentrations 0.4, 0.6 and 0.8 mM, imipramine hydrochloride (Sigma Aldrich) at 0.375 and 0.75 mM or primaquine bisphosphate (Sigma Aldrich) at 1.5 and 3 mM for 30 minutes at 37°C and shaking at 600 rpm. Finally, cells were washed four times with PBS containing 7.5 mM glucose and analyzed by FACS (FACS Calibur, BD).

### Red cell rejuvenation

Where indicated, cells were treated with rejuvesol (100 mM pyruvate, 100 mM inosine, 103 mM phosphate, 5 mM adenine, diluted 1:100 in physiologic buffer supplemented with 7.5 mM glucose) for 1 h at 37 °C prior to experiments.

### LPA stimulation

L-α-lysophosphatidic acid (LPA, Sigma Aldrich, St. Louis, MO, USA) dilution was freshly prepared for every use from a stock solution. Cells were suspended in TBS (10 mM Tris pH 7.5, 150 mM NaCl), 7.5 mM glucose and 1 mM CaCl_2_, stimulated with 1nmol LPA / 5x10^5^ cells and incubated for 15 min at 37 °C with shaking.

### Phosphatidylserine exposure

Stimulated and unstimulated cells, respectively, were washed once with annexin binding buffer (10 mM Hepes pH 7.4, 150 mM NaCl, 5 mM KCl, 1 mM MgCl_2_, 2.5 mM CaCl_2_) and resuspended in annexin binding buffer. AnnexinV-FITC (ImmunoTools, Friesoythe, Germany) was added at a final dilution of 1:100 to the preparations and incubated for 15 min at 4 °C in the dark. After staining, cells were washed once and resuspended in annexin binding buffer for FACS analysis.

### Calcium uptake

Calcium uptake was measured as described by Yang et al. [20]. Briefly, erythrocytes were washed in Hepes buffer (125 mM NaCl, 3 mM KCl, 1 mM MgCl_2_, 2 mM CaCl_2_, 16 mM Hepes pH 7.4, 1.2 mM sodium phosphate, 10 mM glucose). Fluo-3 AM (Invitrogen, Karlsruhe, Germany) was added to a suspension of 2x10^8^ cells/ml with a final concentration of 4 µM Fluo-3 AM. Cells were shaken, protected from light, at 37 °C for 1h. Afterwards, cells were washed three times with TBS (10 mM Tris pH 7.4, 150 mM NaCl) and subjected to LPA stimulation.

### Statistical analysis

The software IBM SPSS statistics 19 was used to perform statistical analyses and to generate plots of the data. 

## Results

In order to characterize and compare functional membrane properties of erythrocytes from NA and NBIA patients, we collected blood from various ChAc and PKAN patients, and from 1 MLS patient. The samples were shipped to Vienna together with simultaneously collected blood samples from unaffected subjects thus functioning as transport controls. In some cases, the samples were used immediately upon arrival for experimental investigations; however, large fractions of all samples were frozen in liquid nitrogen for later analyses. Acanthocytosis was observed in the MLS sample, in all except of one ChAc sample and in six of our PKAN samples. The set of acanthocyte-positive PKAN blood samples is further referred to as PKAN+. As counterpart of our PKAN+ sample set we selected a set of six PKAN samples of similar geographical origin/shipment conditions that did not show acanthocytosis (further referred to as PKAN-). However, genetic testing during the course of the project identified one of these acanthocyte-free samples as MPAN [8] rather than PKAN. A detailed overview of the amount of acanthocytes, the genetic and/or clinical background of the patients is given in Table 1.

**Figure 1:**
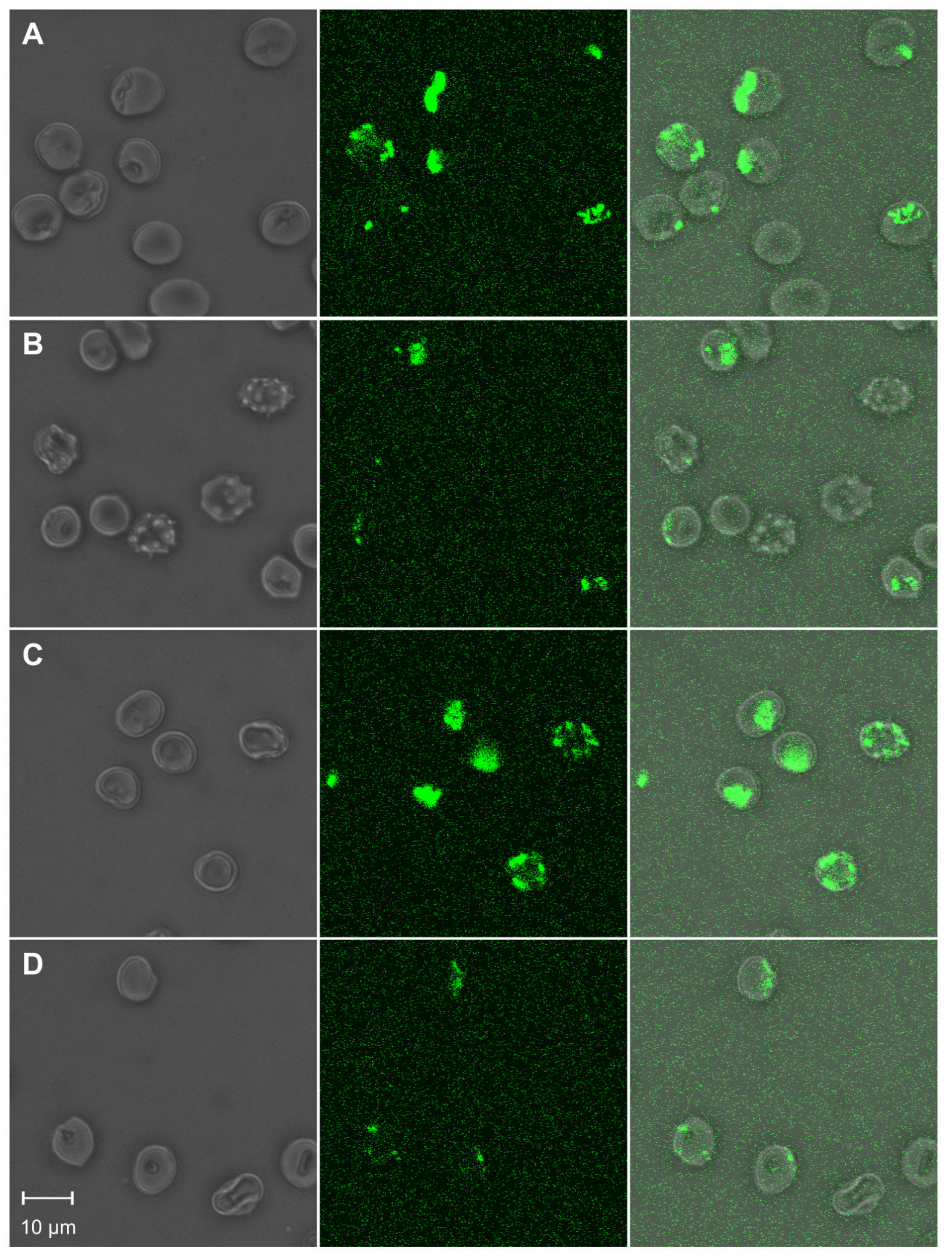
Microscopic comparison of patient’s and control erythrocytes in drug-induced endovesiculation. Erythrocytes of a PKAN+ patient (B and D) and a control donor (A and C) were treated with 3 mM primaquine (A and B) or 0.8 mM chlorpromazine (C and D) in the presence of FITC-dextran to monitor the formation of endovesicles by confocal microscopy. Representative phase contrast (left panels), fluorescence (middle panels) and overlay (right panels) images are shown.

We started our study on alterations of the erythrocyte plasma membrane from NA patients by investigating the effect of the amphiphilic drugs chlorpromazine and primaquine. In normal red cells these drugs induce a stomatocytic shape transformation and the accumulation of endovesicles in the cytosol [21]. Cells were incubated with 0.8 mM chlorpromazine and 3 mM primaquine, respectively, in the presence of FITC-labeled dextran that served as an extracellular fluid phase marker to visualize endovesicle formation. Analyses by phase contrast and fluorescence microscopy revealed that erythrocytes from a PKAN+ patient had significantly less drug-induced endovesicles than control cells (Figure 1). The effect was similar for chlorpromazine and primaquine. Both drugs also induced a similar cell shape transformation from discocyte to stomatocyte in control erythrocytes whereas their effect on the cell shape of the patient’s erythrocytes was dissimilar: 0.8 mM chlorpromazine completely reversed the acanthocytic shape and induced a slight stomatocytic shape in the patient’s cells. In contrast, both stomatocytes and acanthocytes were simultaneously present when the cells were treated with 3 mM primaquine; as expected, endovesicles were only present in stomatocytic cells.

**Figure 2:**
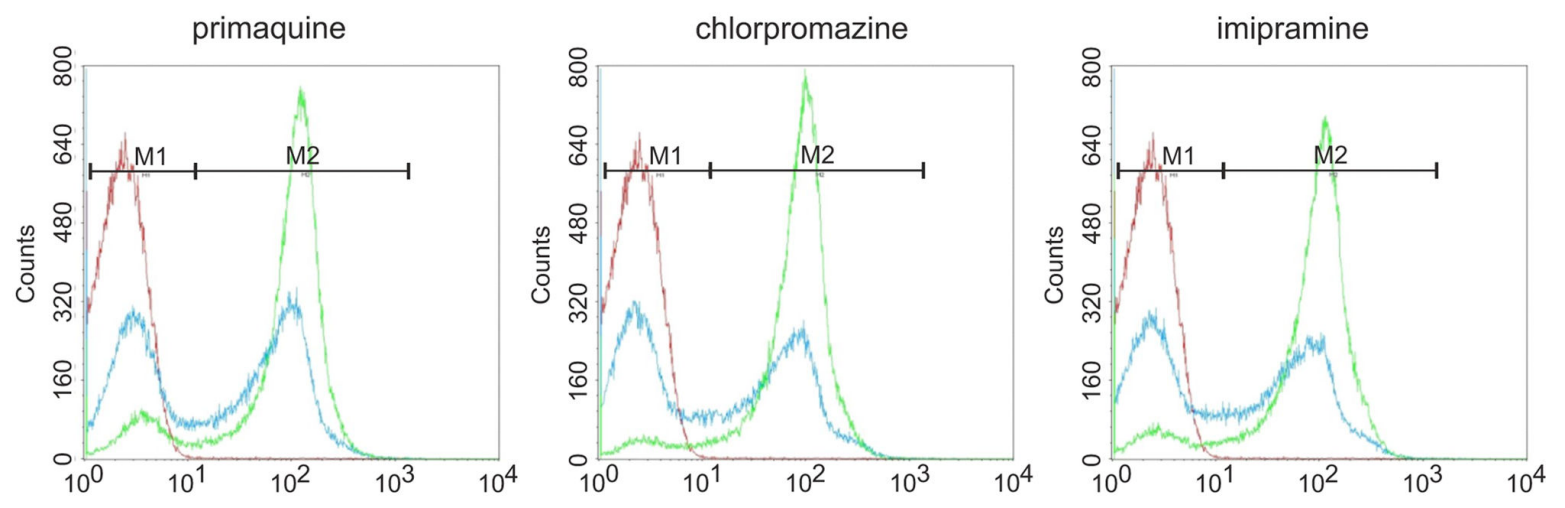
Dose-dependent uptake of fluid phase FITC-dextran by erythrocytes treated with amphiphilic drugs. Erythrocytes were suspended in FITC-labeled dextran and incubated with the indicated amphiphilic drugs to induce endovesiculation. Upon washing, the uptake of fluorescent label was quantified by flow cytometry. Representative histograms are shown. The concentrations were 1.5, 0.4 and 0.375 mM (blue) and 3.0, 0.6 and 0.75 mM (green) for primaquine, chlorpromazine and imipramine, respectively (red is a control incubation without drug).

In order to study the drug-induced endovesicle formation of the whole erythrocyte cell population in a quantitative manner, we developed an assay to detect the uptake of FITC-dextran by flow cytometry. Figure 2 shows the histograms of control erythrocytes that were treated with various concentrations of the drugs chlorpromazine, primaquine and imipramine, respectively. In each case, a highly fluorescent population of cells appears above a certain threshold amount of the amphiphile. This peak of highly fluorescent cells grows at the expense of the unlabeled cell population with increasing concentrations. The concentrations at which these peaks are equally populated are about 0.4 mM for chlorpromazine and imipramine and 1.5 mM for primaquine. This result indicates that endovesicle formation occurs massively above a certain threshold of drug accumulation rather than being a continuous process that linearly depends on the concentration of the drug.

**Figure 3:**
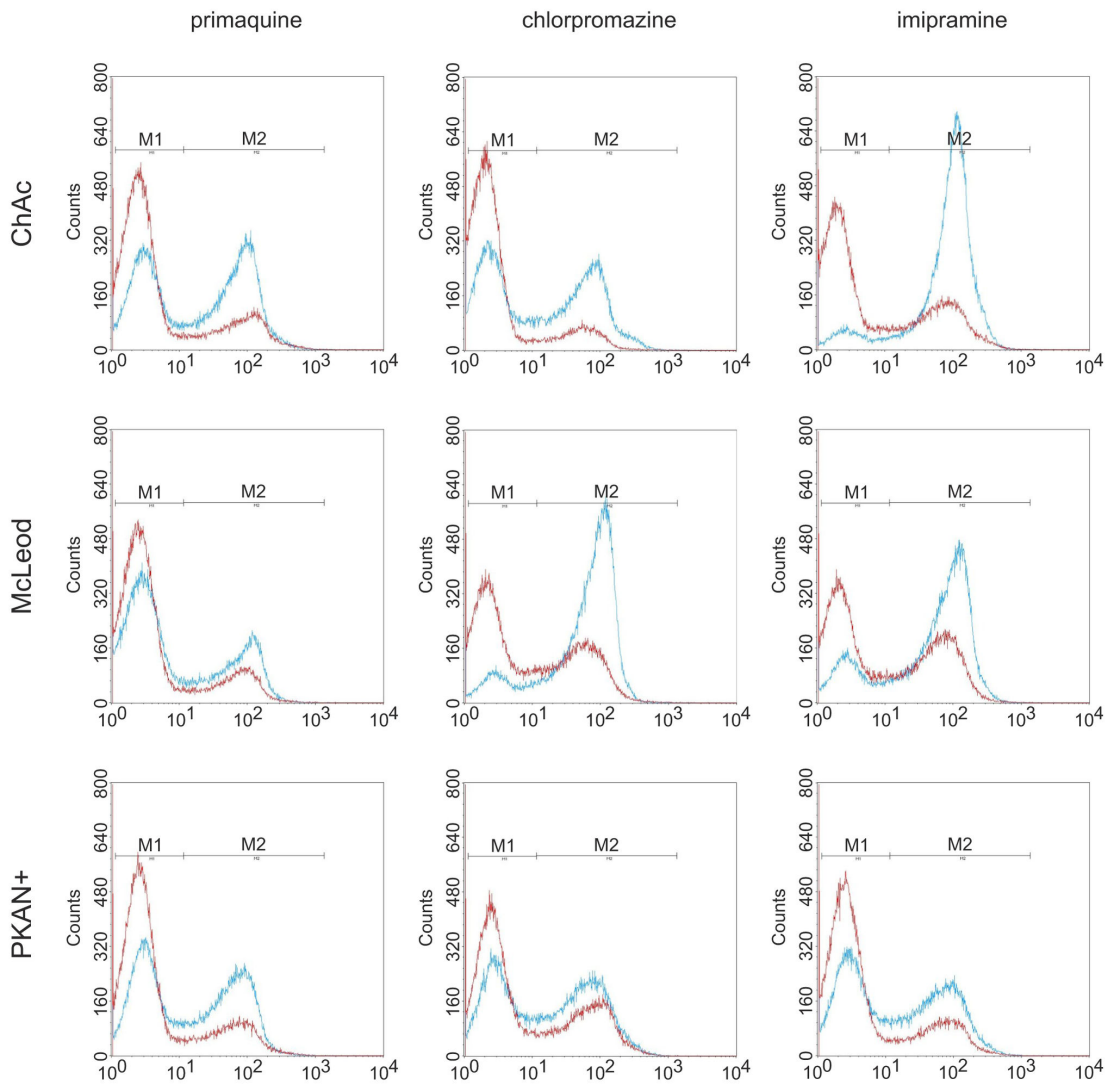
Altered drug-induced endovesiculation of erythrocytes from neuroacanthocytosis patients. ChAc, McLeod and PKAN+, as well as control erythrocytes were subjected to drug-induced endovesiculation using 1.5 mM primaquine, 0.4 mM chlorpromazine and 0.75 mM imipramine. The uptake of FITC-labeled dextran was determined by flow cytometry. Overlays of representative histograms of patients (red) and respective control samples (blue) clearly indicate an impaired ability of the patient’s erythrocytes to form endovesicles.

Next, this flow cytometry assay was applied to study the drug-induced endovesiculation properties of erythrocytes from NA patients in more detail. Patients’ and respective control cells were assayed in parallel for endovesicle formation upon treatment with a given concentration of chlorpromazine, primaquine and imipramine. An overlay of the patients’ and control histograms are shown in Figure 3. Endovesicle formation of ChAc, the MLS and PKAN+ erythrocytes was clearly reduced compared to control cells for all three drugs. This finding is in line with our initial assumption that acanthocytic cell shape and the response to drug-induced endovesiculation are inversely correlated. The difference in the endocytic activity between the control cells in the chlorpromazine and imipramine experiments is likely due to the fact that the ChAc/control and MLS/control samples were assayed immediately upon arrival whereas the PKAN+/control cells had been stored in liquid nitrogen before study.

**Figure 4:**
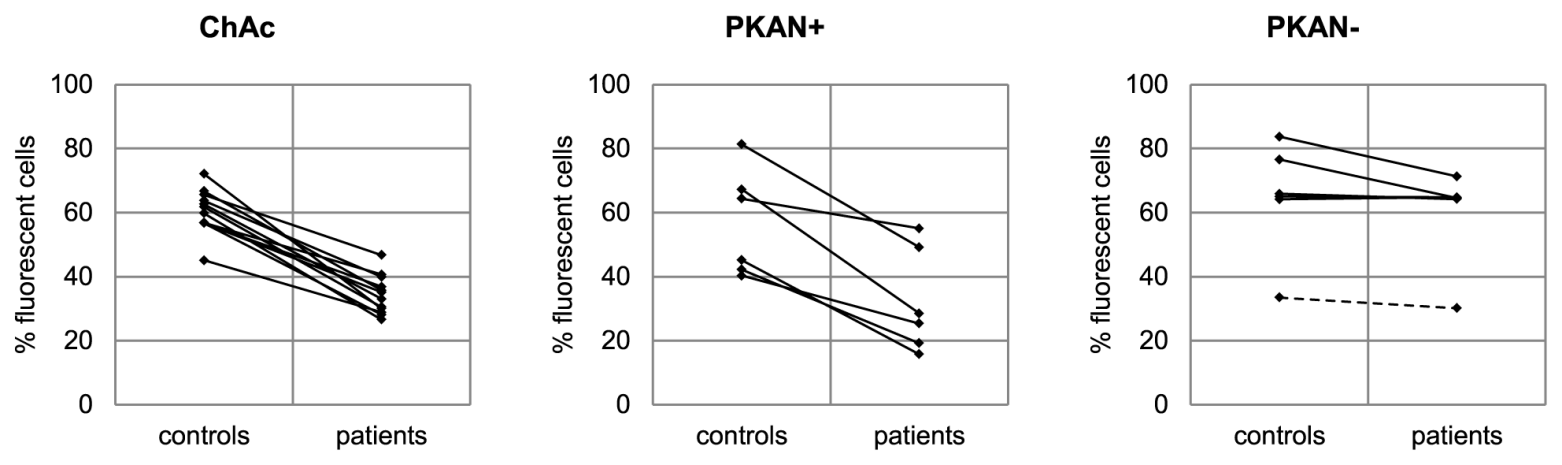
Impaired drug-induced endovesiculation in erythrocytes of patients with acanthocytosis. Erythrocytes from patients (ChAc, PKAN+, PKAN-) and control donors were subjected to drug-induced endovesiculation using 3 mM primaquine. The amount of FITC-dextran positive cells (in %) was assessed by flow cytometry as described. Respective pairs of patient and control donors are connected by lines. The data of the MPAN patient within the NBIA/PKAN- cohort is shown as a dashed line.

**Figure 5:**
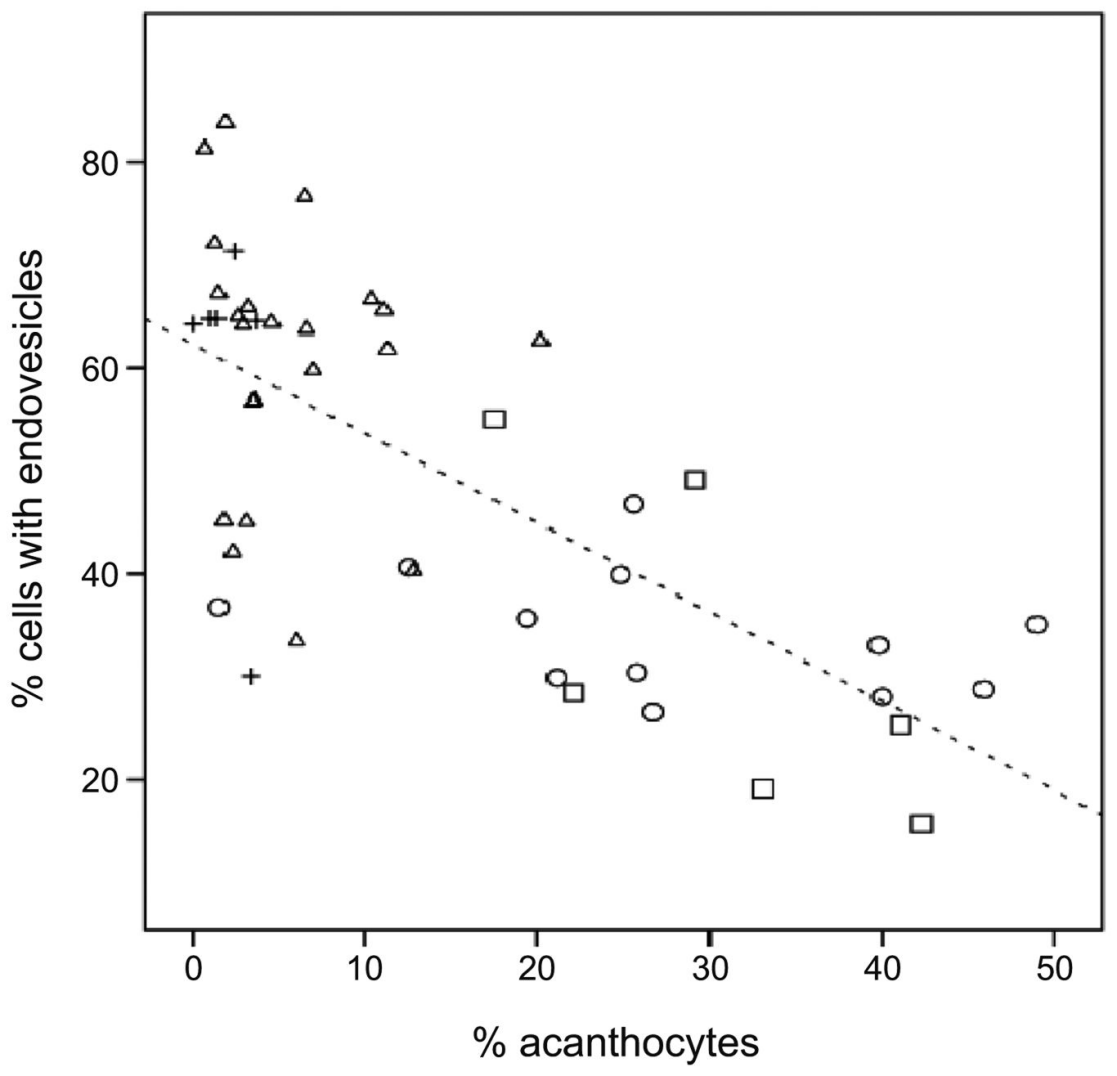
Inverse correlation between drug-induced endovesiculation and erythrocyte shape. The amount of FITC-dextran positive cells upon primaquine-induced endovesiculation (collective data from Figure 4) is blotted against the percent of acanthocytes in the blood sample. ChAc patients (circles), PKAN+ patients (rectangles), PKAN- patients (crosses) and all control samples (triangles) are indicated. A linear regression line is shown (R2 = 0.501). Negative correlation between the ability of erythrocytes to form drug-induced endovesicles and the amount of acanthocytes is observed with Pearson’s r being -0.707 and a 2-tailed significance of 0.01.

In order to obtain a statistically analyzable data set we repeated the measurements with each set of patient-control pairs in parallel. Comparisons of the percentages of FITC-dextran-positive cells (cells in the right region of the histograms) between patients and controls are given in Figure 4 and clearly indicate a reduced endovesiculation activity for erythrocytes from ChAc and PKAN+ patients. In contrast, PKAN- patients are seemingly indifferent from the controls. The statistical analysis of the data by Student’s t-test, given in Table S1, reveals a statistically significant reduction of the amount of cells with endovesicles by about 56% for ChAc and PKAN+ erythrocytes. There is also a slight but statistically insignificant reduction in the fluorescent cell population of PKAN- cells. The data were also analyzed by a paired t-test for differences between each set of patient erythrocyte and the respective transport control cells since differences in the circumstances and durations of the shipment may have affected the cells. Thereby, the significance of the observed differences largely increases for each of the tested NA categories, but it is still not statistically significant (0.098) for PKAN- (Table S1). Analysis of the whole data set for a correlation between the propensity to form endovesicles and the amount of acanthocytes in the blood sample reveals a significant negative correlation with a Pearson’s r of -0.707 (Figure 5).

**Figure 6:**
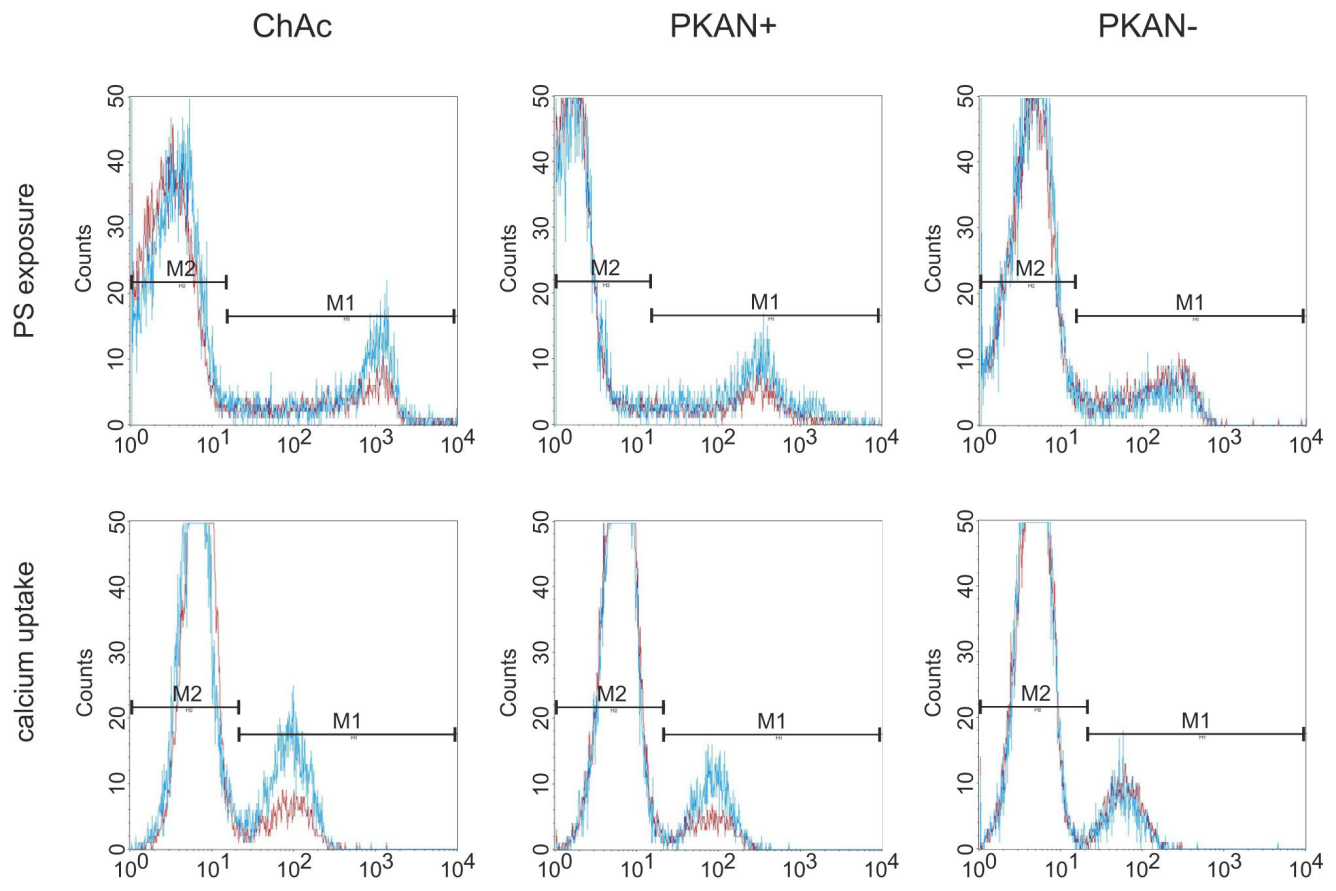
Differences in LPA-induced PS exposure and calcium uptake in erythrocytes of neuroacanthocytosis patients. Erythrocytes from ChAc, PKAN+ and PKAN- patients and control donors were treated with LPA as described in Materials and Methods and stained for PS exposure with FITC-annexin V (upper panels) or calcium uptake with Fluo-3 (lower panels) and analysed by flow cytometry. Overlays of representative histograms of patients (red) and controls (blue) show reduced PS exposure and calcium uptake in both ChAc and PKAN+ samples. The PKAN- sample does not differ from the respective control.

**Figure 7:**
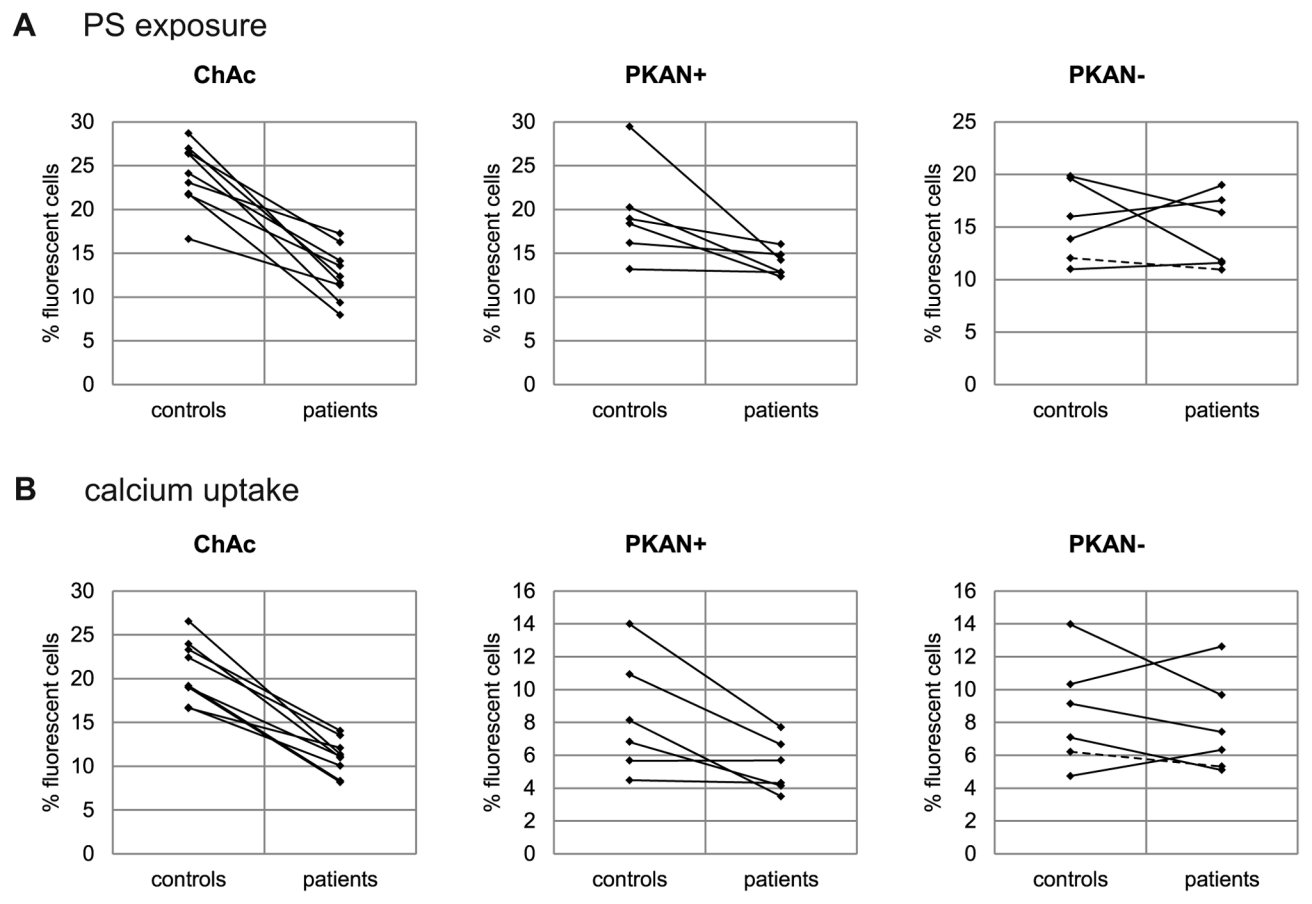
Altered LPA-induced PS exposure and calcium uptake in erythrocytes of patients with acanthocytes. Erythrocytes from patients and control donors were treated with LPA as described in Materials and Methods and either PS exposure (A) or calcium uptake (B) was monitored by flow cytometry. The percentage of FITC-annexin V-positive cells (A) and of Fluo-3-positive cells (B) of patient and control samples are shown and respective pairs are connected by lines. The data of the MPAN patient within the NBIA/PKAN- cohort is shown as a dashed line.

To further characterize differences in the membrane properties of NA erythrocytes we tested their response to LPA. LPA has been reported to open calcium channels [20] in the red cell membrane and to trigger PS exposure, echinocytic shape transformation and exovesiculation [21–23]. Two flow cytometry-based assays were applied using FITC-labeled annexin V to detect exposed PS and Fluo-3 AM - preloaded to the cells - to assess the uptake of calcium, respectively. We observed that upon storage in liquid nitrogen erythrocytes were less responsive to LPA and the results were more variable as compared to fresh cells. However, rejuvenation of the cells after thawing (as described in Materials and Methods) improved the responsiveness of the cells and reduced the variability of the results. Hence rejuvenation was included in the standard procedure for all measurements with the assays described here. The background levels of exposed PS and intracellular calcium in untreated cells was similar for patients’ and control erythrocytes. Interestingly, erythrocytes of a ChAc and a PKAN+ patient showed both reduced PS exposure and calcium uptake upon LPA treatment, whereas the response of cells from a PKAN- patient was again similar to control erythrocytes (Figure 6). Direct comparisons for each patient/control pair of the amount of annexin V-positive cells and Fluo-3 AM-positive cells, respectively, reveal an impaired responsiveness of ChAc erythrocytes to LPA (Figure 7). The effect proves to be statistically highly significant in both a normal two-sample t-test and a paired t-test of differences between patient and the respective transport control (Tables S2 and S3). Again there is clearly no significant difference in the erythrocyte response to LPA for the PKAN- cohort. In contrast, the PKAN+ samples show a reduced response in both LPA-induced PS exposure and calcium uptake but the statistical significance is only shown by one of the t-tests applied, respectively.

**Figure 8:**
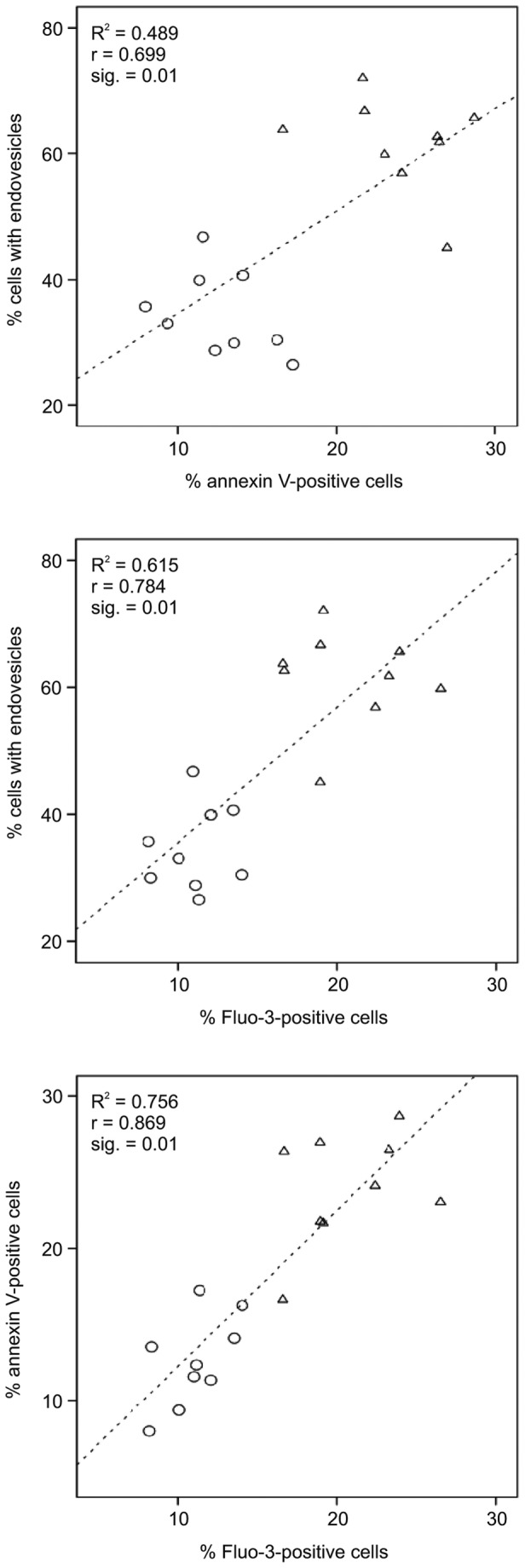
Correlations between drug-induced endovesiculation, LPA-induced PS exposure and calcium uptake. The data of ChAc patients (circles) and respective control samples (triangles) for % of cells with endovesicles upon incubation with primaquine, for % annexin V-positive cells and % Fluo-3-positive cells upon LPA treatment are blotted against each other as indicated (data derived from Figures 4 and 7). Linear regression lines are shown with R2 (shown as inserts). In each combination a positive correlation is observed with Pearson’s r and the 2-tailed significance (given as inserts).

As shown in Figure 7, there is also reduced responsiveness of the PKAN control samples when compared to the ChAc controls, indicating that the transport conditions for these samples were different. It is conceivable that the set of PKAN/control samples is generally in a poorer condition which may obscure a more clear-cut differential effect of the PKAN+ samples in the LPA response assays. Therefore we used only the ChAc/control data set to analyze the correlations between drug-induced endovesiculation, LPA-induced PS exposure and calcium uptake. A highly significant positive correlation is detected in each combination of these variables (Figure 8). It can be concluded that the reduced drug-induced propensity to form endovesicles and the impaired response to LPA with regard to PS exposure and calcium uptake are interdependent features of ChAc (and most probably also PKAN+) erythrocytes and may have a common cause that still needs to be identified.

## Discussion

This study shows that the occurrence of acanthocytes in NA syndromes correlates with alterations in various other properties of the red blood cell membrane, namely LPA-induced PS exposure, calcium uptake and drug-induced endovesiculation. Measurement of these properties may thereby be exploited as tools in addition to microscopic analyses for an early diagnosis of an NA syndrome. In this context it is interesting to note that the ChAc10 sample revealed a reduced endovesiculation property despite the absence of acanthocytosis, thereby indicating that a diminished response in this assay is more predictive for ChAc than the occurrence of acanthocytes. On the other side, regarding our PKAN/NBIA samples, acanthocytosis was a clear predictor of a reduced response in drug-induced endocytosis (Figure 4, Table S1). The inverse correlation between acanthocyte morphology and drug-induced endocytosis can be understood in the light of the bilayer couple hypothesis [16]. Drug-induced endocytosis is based on the relative expansion of the cytoplasmic membrane leaflet by cationic amphiphiles, such as primaquine, chlorpromazine, and imipramine resulting in a stomatocytic cell shape transformation and the formation of endovesicles. The spiked cell shape of acanthocytes indicates a relative expansion of the extracellular membrane leaflet. Hence it is conceivable that less endovesiclulation occurs in acanthocytes than in normal discocytes at a given concentration of the amphiphile. In contrast, our finding of a correlation between reduced drug-induced endovesiculation and impaired LPA-induced PS exposure and calcium uptake in acanthocyte-positive patient samples was surprising (Figure 8) and indicates some interdependence between these alterations. Since the interdependence is yet unknown we refer to these - and maybe other, yet to be discovered aberrations - as the acanthocytic state of NA red blood cells to indicate that acanthocytosis is only one among several correlated aberrations of the red blood cell membrane.

The physiological role of LPA is to induce PS exposure and release of microvesicles from erythrocytes during blood clotting. This involves calcium-dependent and -independent intracellular signaling events [22–24]. LPA induces the opening of calcium channels and inhibits the activity of flippase that maintains the phospholipid asymmetry of the plasma membrane. Both processes can be blocked by protein kinase inhibitors [20,22]. Moreover, it has been shown that PKCζ translocates to the plasma membrane in a LPA-specific manner indicating the involvement of this calcium-independent PKC isoform in the signaling process [22]. In the light of the results presented here, one may assume that red blood cell shape maintenance and pathological shape transformation are regulated processes that influence the LPA-induced signaling and thus lead to a co-appearance of the respective aberrations.

The spectrin-actin cytoskeleton is a prime candidate for which it is conceivable that an erroneous regulation can affect both shape regulation and signaling processes. Alterations of the cytoskeleton have already been described for ChAc erythrocytes. An enrichment of spectrin in the thorns of acanthocytes was observed by scanning and transmission electron microscopy [25,26]. In this respect it is interesting that spectrin has been implicated to be necessary for the regeneration of the normal discoid shape after artificially induced echinocytosis. Shape transformation from echino- to acanthocytes was found under conditions where proper functioning of spectrin was impaired [27]. From this study it can be concluded that the acanthocytic shape is a more stable pathological state than the echinocytic shape. Several studies implicate that echinocytic spikes are devoid of the cytoskeleton [28,29] whereas the cytoskeleton is present in acanthocytic protrusions [27].

Two recent studies on ChAc erythrocytes have shown that alterations in different signaling processes are associated with aberrations in cytoskeletal organization. Foller et al. [30] found altered signaling of PI3K, Rac1 and PAK1 and a higher fraction of depolymerized actin in ChAc erythrocytes indicating an impaired assembly of the junctional complex. DeFranceschi et al. [31] describe increased tyrosine phosphorylation of the cytoskeletal components β-spectrin and adducin and the integral membrane protein band 3 and implicate alterations in the composition of the junctional complex from co-immunoprecipitation studies. Increased phosphorylation and activity of the band 3 anion exchanger in ChAc erythrocytes has already been reported previously [32]. Alterations in cytoskeletal organization can also be inferred for McLeod erythrocytes since the XK-protein, the gene which is mutated in MLS, is directly linked to protein 4.1 which is an essential component of the junctional complex [33]. Moreover, both spectrin and protein 4.1 have been shown to directly bind to the membrane by fatty acid posttranslational modification [34] and/or association with PS [35,36]. Spectrin is thought to be involved in the formation of large PS-rich lipid domains [35] and protein 4.1-deficient red blood cells show alterations in pathways associated with PS-exposure [37]. Thus it is conceivable that the cytoskeleton is also involved in the regulation of membrane phospholipid asymmetry. Moreover, a defect in *PANK2* in terminal erythropoiesis may result in a diminished pool of acyl CoA in mature PKAN+ erythrocytes. Altered neuronal mitochondrial coenzyme A synthesis has also been suggested to be the reason for PKAN [38]. A lack of palmitoyl CoA may lead to defects in palmitoylation-dependent signaling of src family kinases and membrane anchorage of cytoskeletal proteins. Alterations in red cell signaling processes that affect cytoskeletal organization may therefore well be the underlying cause for alterations in membrane shape, drug-induced shape transformation, LPA-induced PS exposure and calcium uptake observed in this study.

The concept that erroneous signaling is causative for the acanthocytic state may also help to understand the conundrum of the occurrence of acanthocytosis in the NA syndromes and the intriguing similarity between ChAc and PKAN+ erythrocytes: (1) different (genetic) causes may have similar effects in a highly interconnected signaling network; (2) small (epi)genetic variations in the conditions of a dynamic signaling system can be sufficient to trigger the transition from a normal to an acanthocytic state [39]; such (yet to be discovered) variations may determine whether or not the acanthocytic state is manifested in erythrocytes of NBIA/PKAN patients.

In addition, several of our findings are noteworth in the light of potential implications for the neuronal side of NA research(1). LPA has been shown to induce cytoskeletal rearrangements in neurons [40]. (2) In nucleated cells, intracellular membranes differ largely in their amount of PS exposed at the cytoplasmic membrane leaflet with high concentrations at the trans-Golgi network and at endosomes [41]. It has already been shown that PS is of importance for vesicles to be transported from the trans-Golgi network to endosomes/lysosomes [42]. Interestingly, this is the process where VPS13A/chorein is thought to play a vital role(3). Moreover, transient redistribution of PS to the outer membrane leaflet has been shown to be associated with calcium-induced exocytosis in neurons and neuroendocrine cells mediated by a calcium-dependent phospholipid scramblase which is essential for the subsequent step of compensatory endocytosis [43]. Together, these findings highlight that LPA signaling and the regulation of phospholipid asymmetry is crucial for the proper functioning of neuronal cells. Further studies on the regulatory changes associated with the acanthocytic state of NA erythrocytes may provide unexpected hints for the molecular aberrations underlying neurodegeneration.

## Supporting Information

Table S1 **Statistical analysis of primaquine-induced endovesiculation.** The numbers are the mean percent values of the FITC-dextran positive cells (derived from Figure 4) upon primaquine treatment (3mM) for each set of patients and control donors and the mean difference of the amount of FITC-dextran positive cells for each pair of patient and control donor (control -patient), respectively (standard deviations are denoted as ±). The data were analyzed by a t-test of means for each set of patients and control donors and a t-test of paired differences for each individual patient-control pair, respectively, and the statistical significances are shown. N gives the number of samples.
(DOCX)

Table S2 **Statistical analysis of LPA-induced PS exposure.** The numbers are the mean percent values of the FITC-annexin V-positive cells (derived from Figure 7) upon LPA treatment for each set of patients and control donors and the mean difference of the amount of FITC-dextran positive cells for each pair of patient and control donor (control -patient), respectively (standard deviations are denoted as ±). The data were analyzed by a t-test of means for each set of patients and control donors and a t-test of paired differences for each individual patient-control pair, respectively, and the statistical significances are shown. N gives the number of samples.
(DOCX)

Table S3 **Statistical analysis of LPA-induced calcium uptake.** The numbers are the mean percent values of the Fluo-3-positive cells (derived from Figure 7) upon LPA treatment for each set of patients and control donors and the mean difference of the amount of FITC-dextran positive cells for each pair of patient and control donor (control -patient), respectively (standard deviations are denoted as ±). The data were analyzed by a t-test of means for each set of patients and control donors and a t-test of paired differences for each individual patient-control pair, respectively, and the statistical significances are shown. N gives the number of samples.
(DOCX)
