# Correction: Alterations of Red Cell Membrane Properties in Neuroacanthocytosis

**DOI:** 10.1371/annotation/9cae31b8-e1d9-4ba8-93e6-1f7276591746

**Published:** 2013-10-24

**Authors:** Claudia Siegl, Patricia Hamminger, Herbert Jank, Uwe Ahting, Benedikt Bader, Adrian Danek, Allison Gregory, Monika Hartig, Susan Hayflick, Andreas Hermann, Holger Prokisch, Esther M. Sammler, Zuhal Yapici, Rainer Prohaska, Ulrich Salzer

The word "Neuroacanthocytosis" is misspelled in the article title. The correct title is: Alterations of Red Cell Membrane Properties in Neuroacanthocytosis. The correct citation is: Siegl C, Hamminger P, Jank H, Ahting U, Bader B, et al. (2013) Alterations of Red Cell Membrane Properties in Neuroacanthocytosis. PLoS ONE 8(10): e76715. doi:10.1371/journal.pone.0076715

