# Supplementary material for: Alterations of Red Cell Membrane Properties in Nneuroacanthocytosis
Source: PLoS One. 2013 Oct 3;8(10):e76715. doi: 10.1371/journal.pone.0076715 (PMC3789665; doi:10.1371/journal.pone.0076715)
Supplement: Table S1 — Statistical analysis of primaquine-induced endovesiculation. The numbers are the mean percent values of the FITC-dextran positive cells (derived from Figure 4) upon primaquine treatment (3mM) for each set of patients and control donors and the mean difference of the amount of FITC-dextran positive cells for each pair of patient and control donor (control -patient), respectively (standard deviations are denoted as ±). The data were analyzed by a t-test of means for each set of patients and control donors and a t-test of paired differences for each individual patient-control pair, respectively, and the statistical significances are shown. N gives the number of samples. (DOCX) [file pone.0076715.s001.docx]

Table S1. Statistical analysis of primaquine-induced endovesiculation

|  |  | N | t-test of means | | t-test of paired differences | |
| --- | --- | --- | --- | --- | --- | --- |
|  |  |  | mean ± st. dev. | significance | control-patient | significance |
| ChAc | controls | 12 | 60.4 ± 6.8 |  |  |  |
| ChAc | patients | 12 | 34.3 ± 6.0 | .000 | 26.0 ± 7.9 | .000 |
| PKAN+ | controls | 6 | 56.8 ± 16.6 |  |  |  |
| PKAN+ | patients | 6 | 32.1 ± 16.2 | .027 | 24.6 ± 11.0 | .003 |
| PKAN- | controls | 6 | 64.8 ± 17.2 |  |  |  |
| PKAN- | patients | 6 | 59.9 ± 14.8 | .611 | 4.9 ± 5.9 | .098 |
