# Supplementary material for: Alterations of Red Cell Membrane Properties in Nneuroacanthocytosis
Source: PLoS One. 2013 Oct 3;8(10):e76715. doi: 10.1371/journal.pone.0076715 (PMC3789665; doi:10.1371/journal.pone.0076715)
Supplement: Table S2 — Statistical analysis of LPA-induced PS exposure. The numbers are the mean percent values of the FITC-annexin V-positive cells (derived from Figure 7) upon LPA treatment for each set of patients and control donors and the mean difference of the amount of FITC-dextran positive cells for each pair of patient and control donor (control -patient), respectively (standard deviations are denoted as ±). The data were analyzed by a t-test of means for each set of patients and control donors and a t-test of paired differences for each individual patient-control pair, respectively, and the statistical significances are shown. N gives the number of samples. (DOCX) [file pone.0076715.s002.docx]

Table S2. Statistical analysis of LPA-induced PS exposure

|  |  | N | t-test of means | | t-test of paired differences | |
| --- | --- | --- | --- | --- | --- | --- |
|  |  |  | mean ± st. dev. | significance | control-patient | significance |
| ChAc | controls | 9 | 24.0 ± 3.7 |  |  |  |
| ChAc | patients | 9 | 12.6 ± 3.0 | .000 | 11.3 ± 4.5 | .000 |
| PKAN+ | controls | 6 | 19.4 ± 5.5 |  |  |  |
| PKAN+ | patients | 6 | 13.8 ± 1.4 | .038 | 5.5 ± 5.5 | .055 |
| PKAN- | controls | 6 | 15.4 ± 3.8 |  |  |  |
| PKAN- | patients | 6 | 14.5 ± 3.5 | .689 | 0.9 ± 4.5 | .655 |
