# Supplementary material for: Alterations of Red Cell Membrane Properties in Nneuroacanthocytosis
Source: PLoS One. 2013 Oct 3;8(10):e76715. doi: 10.1371/journal.pone.0076715 (PMC3789665; doi:10.1371/journal.pone.0076715)
Supplement: Table S3 — Statistical analysis of LPA-induced calcium uptake. The numbers are the mean percent values of the Fluo-3-positive cells (derived from Figure 7) upon LPA treatment for each set of patients and control donors and the mean difference of the amount of FITC-dextran positive cells for each pair of patient and control donor (control -patient), respectively (standard deviations are denoted as ±). The data were analyzed by a t-test of means for each set of patients and control donors and a t-test of paired differences for each individual patient-control pair, respectively, and the statistical significances are shown. N gives the number of samples. (DOCX) [file pone.0076715.s003.docx]

Table S3. Statistical analysis of LPA-induced calcium uptake

|  |  | N | t-test of means | | t-test of paired differences | |
| --- | --- | --- | --- | --- | --- | --- |
|  |  |  | mean ± st. dev. | significance | control-patient | significance |
| ChAc | controls | 9 | 20.7 ± 3.5 |  |  |  |
| ChAc | patients | 9 | 11.1 ± 2.0 | .000 | 9.7 ± 3.2 | .000 |
| PKAN+ | controls | 6 | 8.3 ± 3.5 |  |  |  |
| PKAN+ | patients | 6 | 5.3 ± 1.6 | .089 | 3.0 ± 2.5 | .034 |
| PKAN- | controls | 6 | 8.6 ± 3.3 |  |  |  |
| PKAN- | patients | 6 | 7.7 ± 2.9 | .653 | 0.8 ± 2.5 | .442 |
